# Small nucleolar RNA 42 promotes the growth of hepatocellular carcinoma through the p53 signaling pathway

**DOI:** 10.1038/s41420-021-00740-5

**Published:** 2021-11-10

**Authors:** Ganggang Wang, Jinghua Li, Ye Yao, Yingyi Liu, Peng Xia, Hao Zhang, Maohui Yin, Zhixiang Qin, Weijie Ma, Yufeng Yuan

**Affiliations:** Department of Hepatobiliary and Pancreatic Surgery, Zhongnan Hospital of Wuhan University, 430071 Wuhan, Hubei People’s Republic of China

**Keywords:** Tumour biomarkers, Hepatocellular carcinoma, Targeted therapies, Diagnostic markers

## Abstract

Recent studies show that small nucleolar RNAs (snoRNAs) play an important role in tumorigenesis. SNORA42 is a potential therapeutic target and prognostic biomarker for various cancers, and the aim of the present study was to investigate the function and clinical relevance of SNORA42 in hepatocellular carcinoma (HCC). We detected the expression levels of SNORA42 in HCC and normal liver tissue samples, as well as in tumor and hepatocyte-derived cell lines. SNORA42 was significantly upregulated in the HCC tissues and cells compared to the adjacent liver tissues and normal hepatocytes. Furthermore, overexpression of SNORA42 correlated with poor prognosis in the HCC patients. Knocking down SNORA42 in HCC cell lines decreased their proliferation, migration and invasion in vitro, and inhibited tumor growth and metastasis in vivo. In contrast, ectopic expression of SNORA42 promoted HCC cell proliferation and inhibited apoptosis. Mechanistically, SNORA42 exerted its oncogenic effects by targeting the p53 signaling pathway and cell cycle transition. In conclusion, SNORA42 acted as an oncogene in HCC and was a potential prognostic biomarker and therapeutic target.

## Introduction

Hepatocellular carcinoma (HCC) ranks second in terms of tumor-related deaths and disability-adjusted life years (DALYs) worldwide [1, 2]. At present, surgical resection is the primary treatment for liver cancer [3]. However, since the early stage of HCC is asymptomatic, most patients are initially diagnosed in the advanced stage, which precludes radical surgery [4]. In addition, there are currently limited therapies for the patients without the option for surgery. Therefore, it is crucial to identify novel biomarkers for the early diagnosis and treatment of liver cancer.

Small nucleolus RNAs (snoRNAs) are a class of non-coding RNAs ranging in length from 60 to 300 nucleotides, and are classified into the box C/D snoRNAs and box H/ACA snoRNAs [5]. Recent studies show that the aberrant expression levels of snoRNAs are associated with tumor genesis, development, recurrence, and prognosis. For instance, SNORA42 is highly expressed in non-small-cell lung cancer (NSCLC) tissues and correlates with poor patient prognosis. It also has an oncogenic role in colorectal cancer (CRC), and has been established as a prognostic marker of prostate cancer [6–9]. However, only few snoRNAs that are potentially associated with HCC have been identified so far, and the molecular mechanisms underlying its tumorigenic actions are largely unknown.

We found that SNORA42 was upregulated in HCC tissues and correlated with poor prognosis. In addition, knocking down SNORA42 in HCC cell lines inhibited their proliferation and migration, whereas its ectopic expression had the opposite effect. Furthermore, HCC cells with SNORA42 knockdown showed a significantly impaired ability to form tumors in vivo. Mechanistically, SNORA42 exerted its oncogenic effects by promoting cell cycle transition and preventing apoptosis through the inhibition of the p53 signaling pathway.

## Results

### SNORA42 was overexpressed in HCC tissues and correlates to poor prognosis

**Fig. 1:**
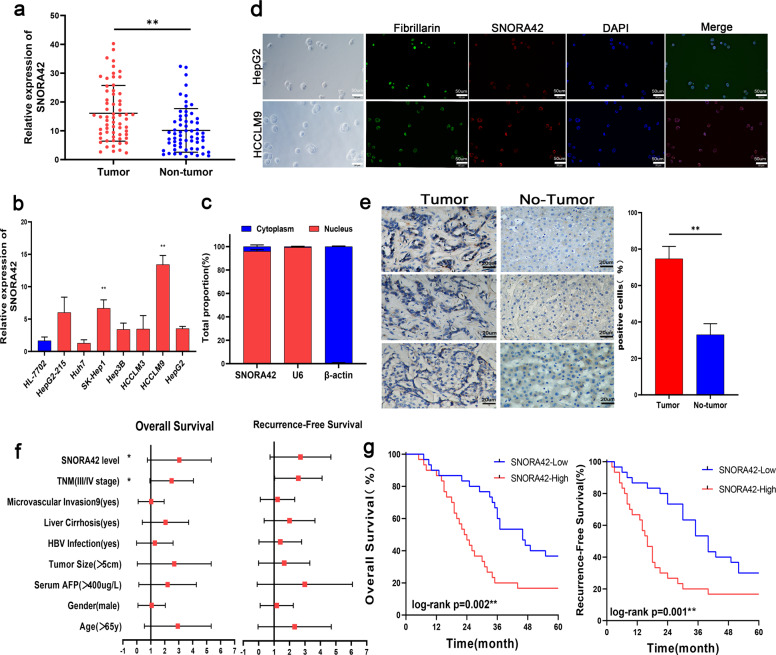
SNORA42 was upregulated in HCC and associated with poor prognosis. **a** SNORA42 expression in the HCC and paired adjacent normal liver tissues. **p < 0.01; Relative expression = 2-ΔΔCT. **b** SNORA42 expression levels in HL-7702 and HCC cell lines. (HCCLM9:11.75 ± 0.8764, *p = 0.0002 and SK-Hep1:5.000 ± 0.8238, *p = 0.0037). **c** qRT-PCR analysis of RNAs purified from nuclear (red) and cytosolic (blue) compartments of HepG2 cells. **d** FISH images showing the expression of SNORA42 in human HCCLM9 and HepG2 cells. (scale bars = 50 µm). **e** Representative ISH images showing in situ expression of SNORA42 in HCC and para-tumor tissues. **f** Forest plot depicting the Cox proportional hazards regression model for overall survival and recurrence-free survival. **g** Kaplan–Meier curves showing overall survival and recurrence-free survival in SNORA42high and SNORA42low patients. (Wilcoxon signed-rank test, overall survival: log-rank **p = 0.002, recurrence-free survival: log-rank **p = 0.001).

SNORA42 was significantly upregulated (*p* < 0.01; Fig. 1a) in the HCC tissues compared to the adjacent liver tissues (tumor margin >3 cm). Consistent with the patient data, SNORA42 was upregulated in all HCC cell lines compared to the immortalized human liver cell line HL-7702 (Fig. 1b), and the expression levels were particularly high in the HCCLM9 (11.75 ± 0.8764, *p = 0.0002) and SK-Hep1 (5.000 ± 0.8238, *p = 0.0037) cells. Furthermore, SNORA42 was detected in the nuclear rather than the cytoplasmic RNA fraction of HepG2 cells (Fig. 1c), and the nuclear localization of SNORA42 was also confirmed by FISH (Fig. 1d). This was consistent with the expression pattern of SNORA42 observed in NSCLC cells18. In addition, the percentage of SNORA42 positive cells were significantly higher in the HCC tissues compared to the adjacent liver tissues (Fig. 1e). Based on the median expression level (from real-time quantitative PCR (qRT-PCR)), the patients were stratified into the SNORA42high and SNORA42low groups. SNORA42 overexpression was significantly associated with microvascular invasion (Table 1; *p = 0.032), which was also confirmed by bivariate correlation analysis (Table 2, *r* = 0.272, *p = 0.035). In addition, high levels of SNORA42 in the tumor tissues predicted shorter survival (log-rank **p = 0.002) and time to recurrence (log-rank **p = 0.001). Finally, multivariate analysis by the Cox proportional hazards regression model for overall survival identified SNORA42 expression level as an independent prognostic risk factor (supplementary materials: Table S2, overall survival: HR = 2.467, 95% CI = 1.094–5.561, *p = 0.029 and Table S3, recurrence-free survival: HR = 2.23, 95% CI = 1.020–4.874, *p = 0.044) (Fig. 1f). Taken together, SNORA42 was a potential biomarker of poor prognosis in HCC patients.

**Table 1:** Relationship between SNORA42 expression and the clinicopathological parameters of HCC patients.

Characteristics		Pantients	SNORA42 level		*p* value
		*N* = 60	Low	High	
Age (y)	<65	22 (36.7)	12	10	0.395
	≥65	38 (63.3)	18	20	
Gender	Male	39 (65.0)	21	18	0.294
	Female	21 (35.0)	9	12	
AFP (μg/L)	<400	25 (41.7)	13	12	0.500
	≥400	35 (58.3)	17	18	
Tumor size (cm)	<5	37 (61.7)	17	20	0.298
	≥5	23 (38.3)	13	10	
HBV infection	No	28 (46.7)	12	16	0.219
	Yes	32 (53.3)	18	14	
Live cirrhosis	No	23 (38.3)	9	14	0.144
	Yes	37 (61.7)	21	16	
Microvascular invasion	No	36 (60.0)	22	14	0.032*
	Yes	24 (40.0)	8	16

**Table 2:** Bivariate correlation analysis between SNORA42 expression and clinicopathological parameters of HCC patients.

Characteristics	SNORA42	
	Spearman correlation	*p*-value
Age (y)	0.069	0.599
Gender	0.105	0.425
AFP (μg/L)	0.034	0.798
Tumor size (cm)	−0.103	0.434
HBV infection	−0.134	0.309
Live cirrhosis	−0.171	0.190
Microvascular invasion	0.272	0.035*
TNM stage	0.237	0.069

### SNORA42 had an oncogenic role in HCC

**Fig. 2:**
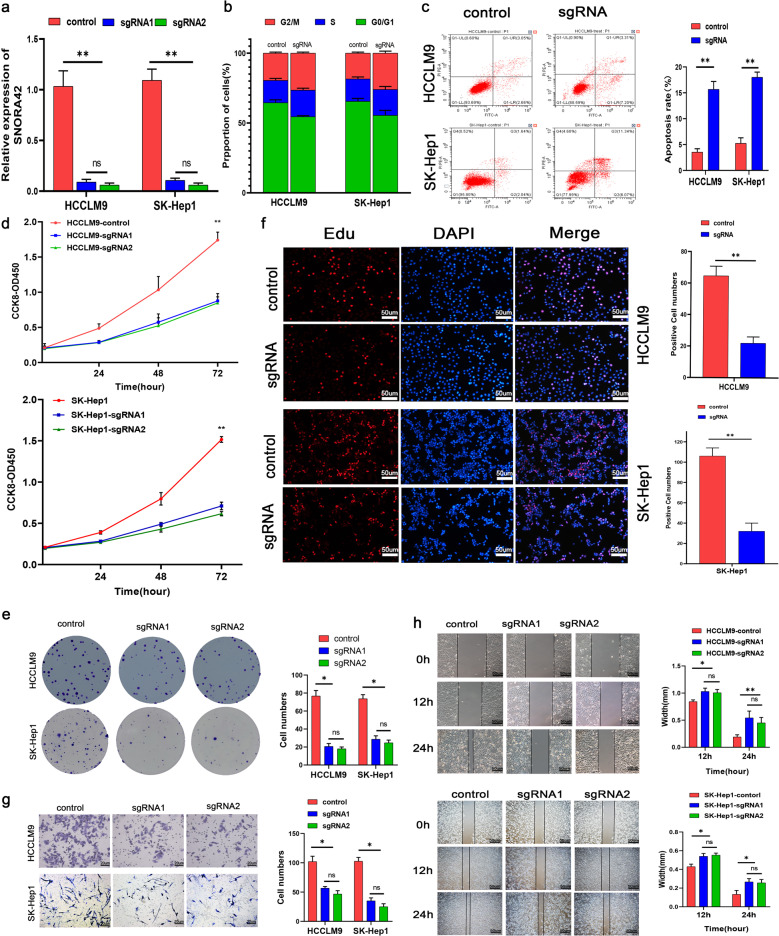
SNORA42 knockdown inhibited HCC cell proliferation, invasion, and migration in vitro. **a** SNORA42 expression level in HCCLM9 and SK-Hep1 cells with SNORA42 knockdown. **b** Flow cytometry plots showing the cell cycle distribution of the indicated groups. **c** Percentage of apoptotic cells in the control and SNORA42-knockdown HCCLM9 and SK-Hep1 cells. **d** Proliferation rates of control and SNORA42-knockdown HCCLM9 and SK-Hep1 cells. **e** Representative images of colonies formed by HCCLM9 and SK-Hep1 cells after SNORA42 knockdown. **f** EdU incorporation in the indicated groups. **g**, **h** Representative images of the Transwell cell invasion assay (**g**) and wound healing assay (**h**) showing the invasion and migration rates of HCCLM9 cells and SK-Hep1 cells after SNORA42 knockdown. *p < 0.05; **p < 0.01.

**Fig. 3:**
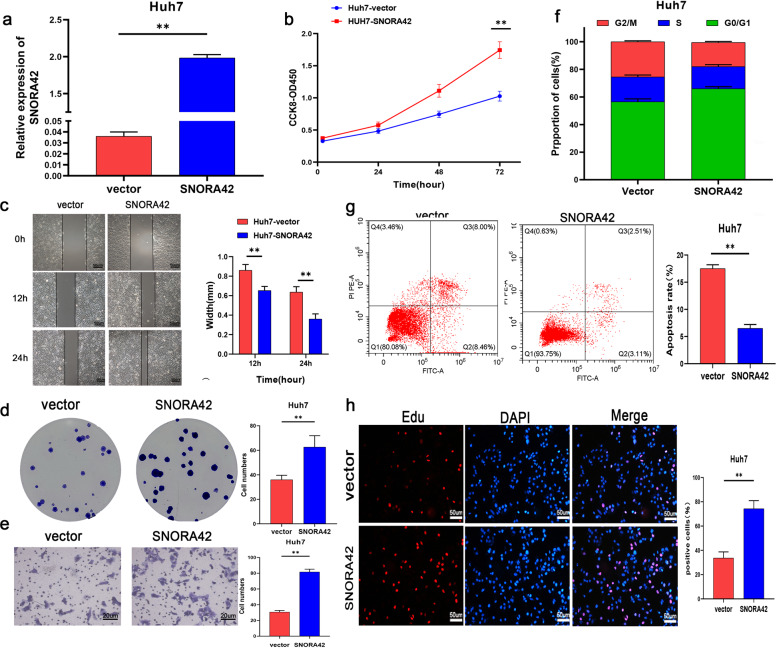
Overexpression of SNORA42 promoted the proliferation of HCC cells and inhibits apoptosis. **a** SNORA42 expression levels in Huh7 cells transfected with pCMV and pCMV-SNORA42. **b** Proliferation rates of control and SNORA42-overexpressing Huh7 cells. **c** Representative images showing extent of wound coverage by the SNORA42-overexpressing Huh7 cells. **d** Representative images of colonies formed by control and SNORA42-overexpressing Huh7 cells. **e** Representative images of the Transwell invasion assay showing the invasion rates of control and SNORA42-overexpressing Huh7 cells. **f** Flow cytometry plots showing the percentage of cells in the different cell cycle stages. **g** Percentage of apoptotic cells in the indicated groups. *p < 0.05, **p < 0.01. **h** EdU incorporation in the indicated groups.

To assess the biological relevance of SNORA42 in HCC, the SK-Hep1 and HCCLM9 cells expressing high levels of SNORA42 were transfected with the target sgRNA sequence14. As shown in Fig. 2a, SNORA42 expression was significantly decreased following gene knockdown (HCCLM9: **p = 0.0038; SK-Hep1: **p = 0.0053).The proportion of SNORA42-knockdown cells in the G0/G1 stage decreased significantly compared to the control, and that in the G2/M stage increased concomitantly (Fig. 2b), indicating cell cycle arrest. SNORA42 silencing also led to a marked increase in the percentage of apoptotic cells (Fig. 2c), along with a reduction in the proliferative capacity of HCC cells, as indicated by the reduced absorbance in CCK8 assay (Fig. 2d), fewer and smaller colonies (Fig. 2e) and lower EdU incorporation (Fig. 2f), compared to the control cells. In addition, knocking down SNORA42 also decreased the in vitro migration and invasion of the HCCLM9 and SK-Hep1 cells (Fig. 2g, h). These results clearly indicated that SNORA42 acted as an oncogene in HCC. To validate this hypothesis, we transfected the SNORA42low Huh7 cells with the overexpression plasmid, which increased SNORA42 levels by a 100-fold compared to the cells transfected with the pCMV vector (Fig. 3a). As expected, SNORA42 overexpression markedly increased the proliferation (Fig. 3b, d, h), migration (Fig. 3c), and invasion (Fig. 3e) of the Huh7 cells, as well as the percentage of cells in the G0/G1 stage compared to the controls, and decreased apoptosis rates (Fig. 3f, g). Taken together, SNORA42 functioned as an oncogene in HCC, and could promote the proliferation, invasion, and metastasis of tumor cells by accelerating cell cycle progression and inhibiting apoptosis.

### SNORA42 expression effected HCC xenograft growth in vivo

**Fig. 4:**
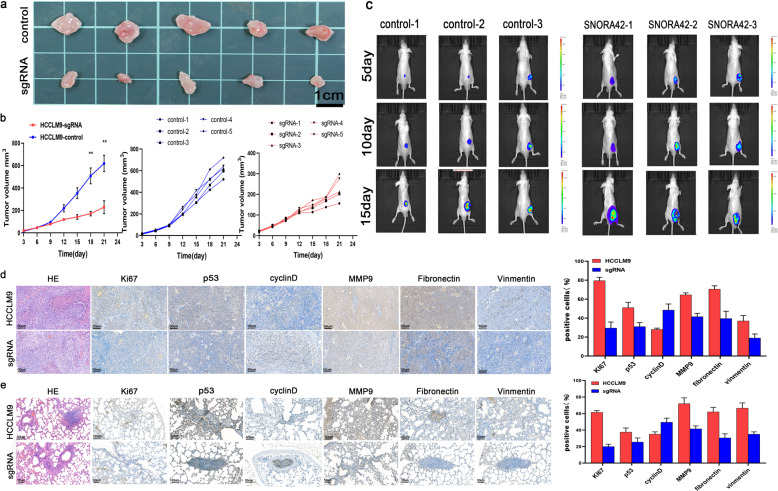
SNORA42 expression in HCC cells effected tumor formation in vivo. **a** Representative images of tumors formed in nude mice injected subcutaneously with the HCCLM9 and HCCLM9-sgRNA cells (*N* = 5). **b** Tumor growth curves in the indicated groups. *p < 0.05, **p < 0.01. **c** Luciferase signals (average wavelength 560 nm) in mice injected with Huh7-vector and pCMV-SNORA42 cells, followed by luciferin. (*N* = 3) **d**, **e** Representative images of HE and immune-stained (Ki67, p53, Cyclin D1, and EMT-related proteins) tumor sections from the HCCLM9 and SNORA42 knockdown groups (scale bars = 50 µm). *p < 0.05, **p < 0.01.

To further establish the oncogenic role of SNORA42, we inoculated nude mice with control and SNORA42-knockdown HCCLM9 cells, and analyzed the xenografts. Compared to the control group, mice injected with SNORA42-knockdown HCC cells displayed slower tumor growth, resulting in visibly smaller tumor volume 21 days after inoculation (Fig. 4a, b). To track tumor growth in vivo, Huh7 cells were additionally transfected with the luciferase gene. Bioluminescence imaging showed that tumor volume in the SNORA42-overexpressing group was significantly higher than that in the control group at all time points (Fig. 4c). We also established a pulmonary metastasis model by injecting the tumor cells through the intravenous route. As shown in Fig. 4e, f, the in-situ expression of Ki67, p53, and epithelial–mesenchymal transition (EMT)-related markers like MMP9, fibronectin, and vimentin were significantly lower in the metastatic nodules and primary tumor tissues of the SNORA42-knockdown versus the control group. Taken together, SNORA42 was essential for HCC progression in vivo and therefore a potential therapeutic target.

### SNORA42 promoted the malignant behavior of HCC cells by inhibiting the p53 pathway

**Fig. 5:**
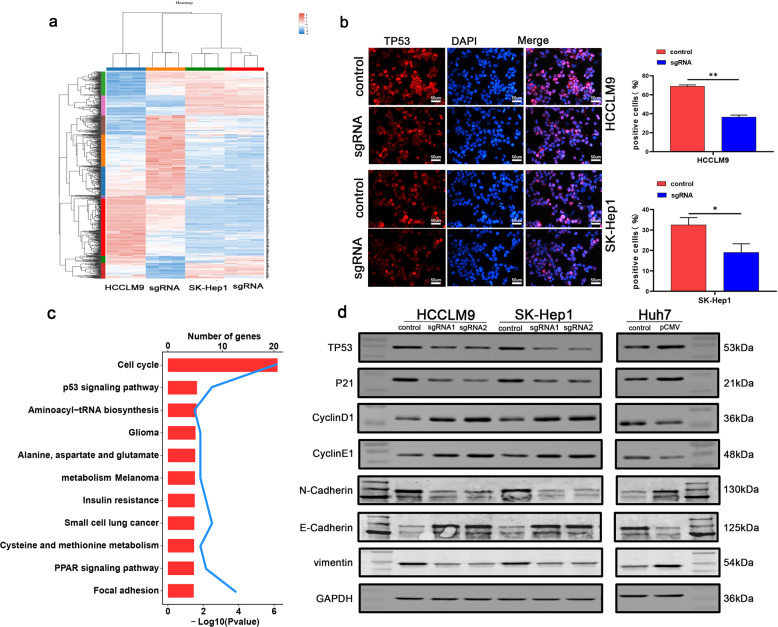
SNORA42 through the p53 signaling signal pathway. **a** RNA-Seq data of the control and SNORA42-knockdown HCCLM9 and SK-Hep1 cells showing the differentially expressed genes. The up- and downregulated genes in the experimental group relative to the control group are shown in shades of red and blue respectively. **b** Representative immunofluorescence images showing the in-situ expression of p53 in HCCLM9 and SK-Hep1 cells after SNORA42 knockdown. *p < 0.05; **p < 0.01. **c** KEGG enrichment analysis showing significant enrichment of cell cycle and p53 signaling pathways among the DEGs. **d** Representative immunoblots showing the expression levels of the indicated proteins in SNORA42-knockdown HCCLM9 and SK-Hep1 cells and SNORA42-overexpressing Huh7 cells.

We identified over 70 differentially expressed genes (DEGs) in the SNORA42-knockdown HCCLM9 and SK-Hep1 cells relative to the controls by RNA sequencing (RNA-Seq) (Fig. 5a). KEGG enrichment analysis showed that the DEGs were significantly enriched in cell cycle and p53 signaling pathways (Fig. 5c), which was similar to that reported for lung cancer cells [18]. Furthermore, immunostaining of the cells showed that the proportion of p53 positive cells was significantly lower in the SNORA42-knockdown group (Fig. 5b). Consistent with this, the SNORA42low Huh7 cells and the HCCLM9 and SK-Hep1 cells with SNORA42 knockdown expressed markedly lower levels of TP53 and P21, and increased levels of Cyclin E and Cyclin D. The opposite results were observed in Huh7 cells overexpressing SNORA42 (Fig. 5d). In addition, vimentin, E-cadherin, and N-cadherin levels were consistent with that observed in animal experiments (Fig. 4d, e). Taken together, SNORA42 promoted HCC progression by inhibiting the p53 signaling pathway.

## Discussion

Liver cancer is a highly invasive malignancy associated with considerable socio-economic burden worldwide [10]. China accounts for almost 50% of the global liver cancer cases as well as related deaths, although there are significant differences between urban and rural areas in terms of incidence and mortality rates [10, 11]. Partial hepatectomy, liver transplantation, radiofrequency ablation (RFA), and transcatheter arterial chemoembolization (TACE) have limited efficacy against HCC [5, 12–14], and the survival of patients is dismal [15]. It is crucial to identify novel markers for the early diagnosis and treatment of inoperable patients.

SRONA42 expression is 1.5-fold higher in lung tumors compared to that in normal lung tissues [16]. Other reports indicate that SNORA42 is also upregulated in prostate cancer [9] and colorectal cancer [8]. Consistent with previous reports, we detected significantly higher expression of SNORA42 in the HCC tissues and cells compared to their normal counterparts, and SNORA42 overexpression correlated with poor prognosis. Furthermore, SNORA42 knockdown in hepatoma cells suppressed their proliferation, invasion, and migration, and inhibited tumor growth in vivo. Studies have implicated SNORA42 in cell cycle progression [6, 8, 9, 17], and we found that SNORA42 knockdown in HCC cells induced cell cycle arrest at the G0/G1 stage. KEGG enrichment analysis further confirmed that the genes differentially expressed in SNORA42-knockdown cells were enriched in p53 signaling pathway and cell cycle.

TP53 is mutated in over 50% of human cancers, and loss of p53 function in cancer cells provides a survival advantage by evading the DNA damage pathway, which allows the aberrant cells to proliferate [18]. The p53 protein is a transcription factor that promotes transcription of genes involved in cell cycle, apoptosis, and immune regulation [19, 20]. P21, a cyclin-dependent kinases inhibitor (CDKI), lies downstream of p53 and is activated in response to DNA damage. It induces the G1 checkpoint arrest to allow the cells to repair the damaged DNA, and thereby prevents the replication and accumulation of damaged DNA [21]. Thus, the p53-p21 axis is an important tumor suppressor, and is frequently disrupted during cancer progression. Regulation of p53 through post-translational modifications, such as phosphorylation, acetylation, ubiquitination, methylation, neddylation, glycosylation, and sumoylation, is crucial for cellular responses to stress [22]. However, the mechanisms underlying SNORA42-mediated regulation of p53 remain to be elucidated.

Nevertheless, a recent study showed that SNORA42-siRNA inhibited lung tumors in mice, which underscores the potential therapeutic relevance of SNORA42 [6], especially for the non-operable cancer patients. In addition, drug resistance arises frequently in liver cancer, which also limits the therapeutic efficacy of conventional chemotherapy [23]. SNORA42 blockade can potentially overcome the problem of drug resistance. Furthermore, SNORA42 can be stably detected in the peripheral blood as well, which makes it an ideal biomarker for tumor diagnosis.

## materials-methodsMaterials and methods

### Patient samples and data

The clinical data of 60 HCC patients (2013–2014) with confirmed pathological diagnosed according to the criteria of the World Health Organization (WHO) were retrieved from the electronic medical records of the Department of Hepatobiliary and Pancreatic Surgery, Zhongnan Hospital. The study was performed according to the Declaration of Helsinki guidelines and was approved by the Ethics Committee of Zhongnan Hospital of Wuhan University (KELUN2020100). All patients provided informed consent. The tissue samples were collected in RNAlater solution, and stored at −80 °C at the Department of Biological Repositories, Zhongnan Hospital, Wuhan University.

### Cell culture

Human HCC cell lines, including Huh7, SK-Hep1, Hep3B, HCCLM3, HCCLM9, HepG2, and HepG2-215, and the immortalized human liver cell line HL-7702 (L02) were purchased from the Center for National Collection of Authenticated Cell Cultures (NCAC, China) and they were recently authenticated and tested for mycoplasma contamination. All cell lines were cultured in high glucose DMEM (Gibco, USA) supplemented with 10% fetal bovine serum (FBS; Gibco, USA) at 37 °C under 5% CO2.

### SNORA42 silencing and overexpression in cell lines

The cells were transfected with SNORA42 sgRNA CRISPR/Cas9 All-in-One Lentivector set (Thermo Fisher Scientific, China) with target sgRNA1 (GACTGGGCAATGGTTCG) and sgRNA2 (CTCACAGCCCACAGGTA) or scrambled sgRNA CRISPR/Cas9 All-in-One Lentivector according to manufacturer’s instructions [8]. As described previously [6, 17], the full-length SNORA42 sequence was cloned into the expression vector p-CMV (Invitrogen, USA) to overexpress SNORA42. Cells were transfected with the SNORA42-pCMV plasmid using lipofectamine 2000 (Life Technologies, USA) according to the manufacturer’s protocol. The expression of SNORA42 was analyzed by qRT-PCR.

### RNA extraction and real-time quantitative PCR

Total RNA was extracted from tissues and cells using Trizol reagent (Invitrogen USA) according to the manufacturer’s protocol, and reverse transcribed using PrimeScript reverse transcription reagent (Takara, Japan). RT-PCR was performed using SYBR Green mix (Toyobo, Japan) on the CFX Connect Real-Time PCR Detection System (Bio-Rad, USA). Each sample was analyzed thrice with β-actin or U6 as the internal control (relative expression = 2-ΔΔCT; ΔCTtest = CTtarget–CTcontrol; ΔΔCT = ΔCTtest–ΔCTcalibrator). The primers were listed in Supplementary Table S1.

### Immunofluorescence

The cultured cells were fixed with 4% paraformaldehyde, permeabilized with 0.5% Triton X-100, and incubated sequentially with primary and secondary antibodies according to the manufacturer’s instructions. After counterstaining with DAPI, the slides were observed under the Olympus FV1000 fluorescence microscope (Tokyo, Japan).

### In situ hybridization

Tissue sections were treated with 3% H2O2 at room temperature for 10 min to inactivate endogenous enzymes, and then boiled in 0.01 M citrate buffer for antigen unmasking. After washing thrice with PBS, the sections were blocked with 5% BSA for 30 min at room temperature, followed by overnight incubation with the primary antibody at 4 °C. The sections were then washed thrice with PBS, incubated with the secondary antibody at 37 °C for 30 min and washed with PBS. Following incubation with SABC for 30 min at 37 °C, the color was developed with DAB for 5–30 min at room temperature. The slides were then immersed in distilled water, counterstained with hematoxylin, and rinsed with running tap water. After dehydration through an alcohol gradient, each section was sealed with 10 µl neutral gum, and observed under a light microscope. The probe sequence was CACTGTGCAACCCCCTTCAGTGCTCACAGCCCACAGGTAAGGGGACTGGGCAATGGTTCGAGGCTGCCATTTGCTATGGCATGGGTACACTATGAGAGGCCCACAGAGAAGGACCCACCATAAATCCATTACCA.

### EdU incorporation assay

The transfected cells were incubated with 50 µM EDU for 2 h, fixed with a commercially available solution at room temperature for 30 min, and then incubated with 2 mg/ml glycine for 5 min. After washing once, the cells were permeabilized with 0.5%TritonX-100 and stained with 200 µl 1 x Apollo staining solution per well. The excess amount of dye was removed by washing with PBS, and the cells were counterstained with DAPI and observed under the fluorescence microscope.

### Cell proliferation assay

Cell proliferation was detected by using the CCK-8 assay and the colony formation assay as previously described [24].

### Wound healing assay

Cell migration was determined using a scratch wound-healing motility assay as previously described [24].

### Transwell invasion assay

Cell invasion was determined using a Transwell invasion assay as previously described [24].

### Flow cytometry

The suitably treated cells were harvested and stained with the Annexin V-FITC/PI cell cycle and apoptosis detection kit (Beyotime Biotechnology, China) according to the manufacturer’s instructions. The stained cells were acquired by flow cytometry (FACSCalibur, BD Biosciences, USA), and the percentage of apoptotic cells and the cell cycle distribution were analyzed.

### Western blotting

The cells were lysed with the RIPA cell lysis buffer containing protease and phosphatase inhibitors (Roche, Germany). The total protein content in the lysates was determined using the BCA kit (Beyotime Biotechnology, China). Equal amounts of protein per sample were separated on a 10% sodium dodecyl sulfate-polyacrylamide (SDS-PA) gel, and transferred to a polyvinylidene fluoride (PVDF) membrane. After blocking with 5% skimmed milk, the membranes were incubated overnight with the primary antibodies (ABclonal, China) at 4 °C. The membrane was washed thrice and incubated with the HRP-conjugated secondary antibody (ABclonal, China) for 1 h. The positive bands were visualized using Clarity Western ECL Substrate (Bio-Rad, USA).

### In vivo experiments

Five-weeks-old male BALB/c nude mice were purchased from the Animal Center of Chinese Academy of Medical Sciences (Beijing) and randomly divided them into the same two groups for subsequent experiments. HCC cells (1 × 106 cell/mouse) were inoculated subcutaneously into each mouse to establish xenografts. The length and width of the palpable tumors were measured with Vernier caliper every 3 days, and the volume (*V* = 0.52L × W × W) was calculated. After 3 weeks, the mice were euthanized and the tumor tissues and major organs were examined. For metastasis induction, the mice were inoculated with 1 × 105 cell/ml hepatoma cells in 200 µl media through the intravenous route. The body weight of the mice was recorded regularly, and the mice were euthanized 5 weeks after inoculation with an intraperitoneal injection of 2% sodium pentobarbital 300 μl (215 mg/kg). For in vivo imaging, luciferin 200 μl (0.5 mg/ml) was injected into the caudal vein, and luciferase signals were measured at 560 nm using a multispectral living imaging system (PerkinElmer IVIS Lumina LT Series III, USA).

### Statistical analysis

All data were expressed as mean ± standard deviation (mean ± SD). Statistical analysis was performed using SPSS20. Survival rate was calculated by the Kaplan–Meier method and Cox proportional hazard regression model, and compared by the logarithmic rank sum test. *P* values < 0.05 were considered statistically significant.

### RNA-sequencing

RNA integrity was assessed using the Agilent 2100. Sequencing libraries were generated using VAHTS mRNA-seq v2 Library Prep Kit for Illumina. The libraries were sequenced on an Illumina NovaSeq platform according to the manufacturer’s instructions. Differentially expressed genes were defined on the basis of *P*-value < 0.05 and absolute log2 fold change > 1 (Peng et al. 24). The data were analyzed on the free online platform of Majorbio Cloud Platform (www.majorbio.com).

## Supplementary information

Table S1

Table S2

Table S3

## Data Availability

The datasets used and/or analyzed during the current study are available from the corresponding author on reasonable request.
