# Supplementary material for: Small nucleolar RNA 42 promotes the growth of hepatocellular carcinoma through the p53 signaling pathway
Source: Cell Death Discov. 2021 Nov 10;7:347. doi: 10.1038/s41420-021-00740-5 (PMC8581050; doi:10.1038/s41420-021-00740-5)
Supplement: Supplementary file 1 — Table S1 [file 41420_2021_740_MOESM1_ESM.docx]

**Table S1: Primer sequences used in this study.**

| Primer name | Primer sequence |
| --- | --- |
| β-actin forward | CTCCATCCTGGCCTCGCTGT |
| β-actin reverse | GCTGTCACCTTCACCGTTCC |
| U6 forward | GCTTCGGCAGCACATATACTAAAAT |
| U6 reverse | CGCTTCAAATTTGCGTGTCAT |
| SNORA42 forward | TGGATTTATGGTGGGTCCTTCTCTG |
| SNORA42reverse | CAGGTAAGGGGACTGGGCAATGGTT |
