# Supplementary material for: Small nucleolar RNA 42 promotes the growth of hepatocellular carcinoma through the p53 signaling pathway
Source: Cell Death Discov. 2021 Nov 10;7:347. doi: 10.1038/s41420-021-00740-5 (PMC8581050; doi:10.1038/s41420-021-00740-5)
Supplement: Supplementary file 2 — Table S2 [file 41420_2021_740_MOESM2_ESM.docx]

**Table S2: Univariate and multivariate analyses of clinicopathological parameters about overall survival.**

| Characteristics | Univariate analysis | | | Multivariate analysis | | |
| --- | --- | --- | --- | --- | --- | --- |
|  | p | HR | 95%CI | p | HR | 95%CI |
| Age(y） | 0.140 | 1.632 | 0.851-3.129 | 0.435 | 1.642 | 0.473-5.695 |
| Gender | 0.299 | 1.383 | 0.750-2.551 | 0.606 | 0.760 | 0.268-2.157 |
| AFP(ug/L) | 0.288 | 1.396 | 1.754-2.586 | 0.867 | 0.867 | 0.164-4.575 |
| Tumor Size(cm) | 0.220 | 1.455 | 0.799-2.648 | 0.306 | 1.811 | 0.581-5.651 |
| HBV infection | 0.585 | 1.183 | 0.647-2.162 | 0.787 | 0.850 | 0.262-2.761 |
| Live Cirrhosis | 0.784 | 1.090 | 0.589-2.016 | 0.958 | 0.963 | 0.234-3.961 |
| Microvascular Invasion | 0.060 | 1.777 | 0.977-3.233 | 0.570 | 0.745 | 0.269-2.062 |
| TNM stage | 0.003** | 2.645 | 1.346-4.515 | 0.021* | 2.168 | 1.123-4.186 |
| SNORA42 Level | 0.006** | 2.37 | 1.288-4.362 | 0.029* | 2.467 | 1.094-5.561 |

**HR=Hazard Ratio; 95%CI= 95% confidence interval.**
