# Supplementary material for: Small nucleolar RNA 42 promotes the growth of hepatocellular carcinoma through the p53 signaling pathway
Source: Cell Death Discov. 2021 Nov 10;7:347. doi: 10.1038/s41420-021-00740-5 (PMC8581050; doi:10.1038/s41420-021-00740-5)
Supplement: Supplementary file 3 — Table S3 [file 41420_2021_740_MOESM3_ESM.docx]

**Table S3: Univariate and multivariate analyses of clinicopathological parameters about recurrence-free survival.**

| Characteristics | Univariate analysis | | | Multivariate analysis | | |
| --- | --- | --- | --- | --- | --- | --- |
|  | p | HR | 95%CI | p | HR | 95%CI |
| Age(y） | 0.067 | 1.828 | 0.959-3.484 | 0.485 | 1.527 | 0.466-5.005 |
| Gender | 0.343 | 1.339 | 0.732-2.450 | 0.749 | 0.845 | 0.302-2.367 |
| AFP(ug/L) | 0.140 | 1.583 | 0.861-2.909 | 0.836 | 1.197 | 0.219-6.549 |
| Tumor Size(cm) | 0.344 | 1.329 | 0.737-2.397 | 0.869 | 1.103 | 0.345-3.522 |
| HBV infection | 0.341 | 1.336 | 0.737-2.422 | 0.924 | 0.946 | 0.303-2.957 |
| Live Cirrhosis | 0.545 | 1.206 | 0.657-2.215 | 0.889 | 0.901 | 0.209-3.894 |
| Microvascular Invasion | 0.063 | 1.745 | 0.970-3.137 | 0.816 | 0.885 | 0.317-2.470 |
| TNM stage | 0.001** | 2.699 | 1.490-4.887 | 0.011* | 2.269 | 1.205-4.273 |
| SNORA42 Level | 0.009** | 2.211 | 1.223-3.996 | 0.044* | 2.230 | 1.020-4.874 |

**HR=Hazard Ratio; 95%CI= 95% confidence interval.**
